# Reliability of overbite depth indicator (ODI) and anteroposterior dysplasia indicator (APDI) in the assessment of different vertical and sagittal dental malocclusions: a receiver operating characteristic (ROC) analysis

**DOI:** 10.1590/2177-6709.21.5.075-081.oar

**Published:** 2016

**Authors:** Farheen Fatima, Mubassar Fida, Attiya Shaikh

**Affiliations:** 1 Resident in Orthodontics, The Aga Khan University Hospital, Section of Dentistry, Department of Surgery, Karachi Pakistan.; 2 Consultant Orthodontist/Associate Professor, The Aga Khan University Hospital, Program Director Orthodontics Residency Program, Section of Dentistry, Department of Surgery, Karachi, Pakistan.; 3 Consultant Orthodontist/ Assistant Professor, The Aga Khan University Hospital, Program Coordinator, Orthodontics Residency Program, Section of Dentistry, Department of Surgery, Karachi, Pakistan.

**Keywords:** Malocclusion, Overbite, Cephalometry, Receiver operating characteristic curve

## Abstract

**Introduction:**

Differential diagnosis of skeletal and dental relationships is crucial for planning orthodontic treatment. Overbite depth indicator (ODI) and anteroposterior dysplasia indicator (APDI) had been introduced in the past for assessment of vertical and sagittal jaw relationships, respectively.

**Objective:**

The objectives of this study were to evaluate the reliability of ODI and APDI in overbite and Angle malocclusions, as well as assess their diagnostic reliability among males and females of different age groups.

**Material and Methods:**

This study was conducted using pretreatment dental casts and lateral cephalograms of 90 subjects. For ODI, subjects were divided into three groups based on overbite (normal overbite, open bite and deep bite). Likewise, the same subjects were divided for APDI into three groups, based on Angle's malocclusion classification (dental Class I, II and III malocclusions). Mann-Whitney U test was applied for comparison of study parameters regarding sex and different age groups. The mean values of ODI and APDI were compared among study groups by means of Kruskal-Wallis and post-hoc Dunnet T3 tests. The receiver operating characteristic (ROC) curve was applied to test diagnostic reliability.

**Results:**

Insignificant differences were found for ODI and APDI angles, particularly in regards to sex and age. Significant intergroup differences were found in different overbite groups and Angle's classification for ODI and APDI, respectively (*p* < 0.001). ROC showed 91% and 88% constancy with dental pattern in ODI and APDI, respectively.

**Conclusions:**

ODI can reliably differentiate deep bite *versus* normal overbite and deep bite *versus* open bite. APDI can reliably differentiate dental Class I, II and III malocclusions.

## RESUMO

**Introdução:**

o diagnóstico diferencial das relações esqueléticas e dentárias é essencial para o plano de tratamento ortodôntico. O indicador de profundidade da sobremordida (ODI)) e o indicador de displasia anteroposterior (APDI) foram desenvolvidos, no passado, para avaliação das relações verticais e sagitais dos maxilares, respectivamente.

**Objetivo:**

o objetivo desse estudo foi avaliar a confiabilidade do ODI e do APDI em diferentes sobremordidas e más oclusões de Angle, bem como verificar sua confiabilidade diagnóstica em homens e mulheres de diferentes grupos etários.

**Métodos:**

esse estudo foi conduzido utilizando-se modelos de estudo e radiografias cefalométricas laterais pré-tratamento de 90 indivíduos. Para o ODI, os indivíduos foram divididos em três grupos, com base na sobremordida (sobremordida normal, mordida aberta, mordida profunda). Ainda, para avaliação do APDI, esses mesmos indivíduos foram divididos em três grupos baseados na classificação de Angle para as más oclusões (Classes I, II e III dentárias). O teste *U* de Mann-Whitney foi aplicado para comparar os parâmetros estudados, quanto ao sexo e diferentes grupos etários. Os valores médios do ODI e do APDI foram comparados entre os grupos estudados por meio dos testes de Kruskal-Wallis e *post-hoc* T3 de Dunnett. A curva ROC (*receiver operating characteristic*) foi aplicada para testar a confiabilidade do diagnóstico.

**Resultados:**

diferenças não significativas foram encontradas para os ângulos ODI e APDI, particularmente em relação ao sexo e à idade. Diferenças significativas foram encontradas entre os grupos com diferentes sobremordidas e diferentes más oclusões de Angle, para o ODI e para o APDI, respectivamente (*p* < 0,001). A ROC mostrou 91% e 88% de concordância com o padrão dentário, para o ODI e APDI, respectivamente.

**Conclusões:**

o ODI mostrou-se confiável para diferenciar entre a mordida profunda e a sobremordida normal, e entre a mordida profunda e a mordida aberta. O APDI é confiável para se diferenciar entre as más oclusões dentárias de Classes I, II e III.

## INTRODUCTION

Malocclusions are classified on the basis of skeletal discrepancies and occlusal disharmonies. In clinical practice, a dental malocclusion is usually found with a corresponding skeletal discrepancy. However, in several cases, dental and skeletal malocclusions may not follow an analogous pattern. This might be due to variations in dental malocclusion which are more amenable to environmental influences.^1^ Hence, differential diagnosis is crucial for planning the treatment of complex orthodontic problems. 

Identification of dentoalveolar and skeletal relationships in the vertical and sagittal planes can be achieved by various cephalometric analyses.^2^ ^-^ ^6^ Skeletal relationship in the vertical plane is commonly assessed by Downs mandibular plane angle (FMA), Y-axis, Steiner mandibular plane angle (SNMP), facial angle and several others. In 1948, Downs^4^ introduced FMA, Y-axis and facial angle, using Frankfort horizontal plane as the reference plane. The problem regarding these analyses was related to difficulty identifying the landmarks. Additionally, the mandibular plane used in FMA was drawn as a tangent to the lower border of the body of the mandible, which is not very reliable and may lead to measurement error.^5^ To overcome this deficiency and facilitate diagnosis, Kim^7^ studied cephalograms of 119 subjects with ideal occlusion and 500 subjects with different malocclusions, and introduced the overbite depth indicator (ODI) to assess the skeletal relationship in the vertical plane. The ODI is the sum of two interplaner angles that showed the highest correlation with incisor overbite. It describes the skeletal trends towards open bite or deep bite.

Assessment of sagittal skeletal relationship is most commonly performed by ANB angle, Wits appraisal, McNamara analysis and several others.^2^ ^,^ ^3^ ^,^ ^8^ ^,^ ^9^ Riedel^2^ introduced the ANB angle in 1952. It estimates the discrepancy of maxilla and mandible in reference to the anterior cranial base. Various studies have reported that the values of the ANB angle are affected by steepness of the S-N plane, variation in the position of point A due to root position, exceptionally long or short mandible, and excessively long or short face.^3^ ^,^ ^6^ To overcome these problems, Jacobson,^3^ in 1975, proposed a simple method to measure the degree of anteroposterior dysplasia: "Wits appraisal." In this method, perpendicular lines were drawn from points A and B on the occlusal plane. However, the value of Wits appraisal was affected by occlusal plane angle and incisor angulations.^6^ Moreover, these analyses do not describe the relationship between dental and skeletal patterns. Hence, the diagnosis drawn from the most commonly used analyses is still questionable.^2^ ^,^ ^3^ ^,^ ^8^ ^,^ ^9^ ^,^ ^10^ In order to overcome these shortcomings, Kim and Vietas^11^ studied cephalograms of 102 subjects with normal occlusion and 874 subjects with different dental malocclusions based on Angle's classification, and proposed the anteroposterior dysplasia indicator (APDI) which scores the sagittal skeletal relationship. The APDI is the sum of three interplaner angles that showed the highest correspondence with Angle's classification.^12^ ^,^ ^13^ ^,^ ^14^

Similarly, a few studies have been conducted to test the reliability of ODI and APDI in Caucasian and Japanese populations.^7^ ^,^ ^11^ ^,^ ^14^ However, to date, no study has been conducted in a Pakistani population. Therefore, the aim of our study was to determine and compare the mean ODI and APDI values in various overbite and Angle's classification groups, respectively. In addition, we aimed to assess the diagnostic validity of ODI and APDI and compare them among different sex and age groups.

## MATERIAL AND METHODS

Data were collected retrospectively from the pretreatment orthodontic records of patients presenting to our dental clinics during 2006-2015. Sample size was calculated using the values of ODI in three overbite groups, as reported by Freudanthaler et al.^12^ Alpha was set as 0.05 and the power of study as 80% for sample size calculation which showed that a sample of 16 was necessary in each group. However, to ensure the validity of comparison among different study groups, sample size was increased to 30 subjects in each of the three groups. 

Subjects with good-quality pretreatment lateral cephalograms and dental casts with well-established molar and incisor relationship were included in the study. A digital vernier caliper (0-150 mm ME00183, Dentaurum, Pforzheim, Germany) with accuracy of 0.02 mm and reliability of 0.01 mm (manufacturer's specification) was used to record overbite on dental casts. Subjects having subdivision malocclusion and those with anterior teeth showing combined characteristics of open and deep bite were excluded. 

A sample of 90 subjects was divided into three groups for ODI on the basis of overbite:

» Normal overbite group: overbite 1-3 mm (30 subjects);
» Open bite group: overbite < 0 mm (30 subjects);
» Deep bite group: overbite > 4 mm (30 subjects).

For APDI, 90 subjects were equally divided into three groups on the basis of Angle's classification of malocclusion^8^:

» Dental Class I (30 subjects);
» Dental Class II (30 subjects);
» Dental Class III (30 subjects).

**Figure 1:**
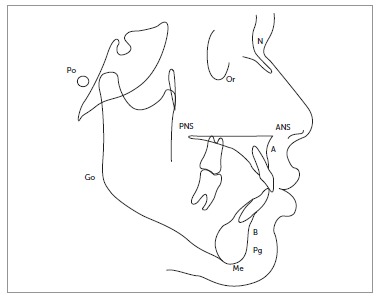
Landmarks for ODI and APDI.

Each study group was further divided into adolescent group (10-18 years old) and adult group (19-30 years old). Each group included 14 males and 16 females, except for the open bite group that had an equal number of male and female subjects. Lateral cephalograms of these subjects were traced manually on acetate paper, with a 0.5-mm lead pencil in a dark room by the main investigator. Specific landmarks were identified (N, Or, Po, ANS, PNS, A, B, Pg, Me, Go) and angular measurements were determined with the aid of a protractor (Fig 1).

**Figure 2:**
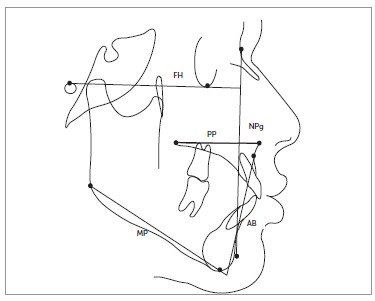
ODI and APDI parameters.

The ODI was measured as the sum of two angles (AB-MP and PP-FH), as described by Kim^7^ (Fig 2).

The APDI was measured as the sum of three angles (FH-NPg, PP-FH and AB-NPg), as described by Kim and Vietas^11^ (Fig 2). 

Statistical analysis of data was carried out by means of SPSS for Windows (version 20.0, SPSS Inc. Chicago, USA). Shapiro-Wilk test was used to test for normality of data and revealed non-normal distribution; hence, non-parametric tests were applied. Mann-Whitney-U test was used to compare the study parameters between males and females as well as adolescent and adult groups. The mean values of ODI and APDI angles were compared among study groups by means of Kruskal-Wallis test. Multiple comparisons for ODI and APDI among study groups were carried out by means of post-hoc Dunnet T3 test. A *p* ≤ 0.05 was consigned as statistically significant. The reliability of ODI and APDI as diagnostic analyses was tested by means of the receiver operating characteristic curve (ROC).

## RESULTS

The study parameters were compared between males and females as well as between adolescents and adults. Results showed insignificant differences. Hence, to conserve the power of study, data were not stratified according to sex and age (Tables 1 and 2). 

**Table 1:** Comparison of study parameters between adolescent and adult groups.

Parameters	Adolescents (n = 44)		Adults (n = 46)		*p*-value
	Median	Range	Median	Range	
Over bite (mm)	3.00	10.5 (-4.0 to 6.5)	3.00	16.5 (-6.5 to 10.0)	0.607
AB-MP (degrees)	71.50	40 (49 to 89)	74.00	38 (53 to 91)	0.824
PP-FH (degrees)	2.00	15 (-6 to 9)	2.00	18 (-7 to 11)	0.987
ODI (degrees)	74.00	43 ( 54 to 97)	73.50	45 (53 to 98)	0.929
FH-NPg (degrees)	87.00	22 (78 to 100)	88.00	17 (79 to 96)	0.682
AB-NPg (degrees)	-7.00	22 (-16 to 6)	-6.00	24 (-15 to 9)	0.382
APDI (degrees)	83.50	46 (62 to 108)	83.00	45 (65 to 110)	0.492

n = 90. Mann-Whitney U Test. *p* ≤ 0.05.

Comparison among overbite groups showed significant differences for AB-MP angle (*p* < 0.001) and ODI (*p* < 0.001). However, insignificant difference was found for the palatal plane angle among the three overbite groups (*p* = 0.775) (Table 3).

**Table 2:** Comparison of study parameters between male and female groups.

Parameters	Male (n = 44)		Female (n = 46)		*p*-value
	Median	Range	Median	Range	
Over bite (mm)	2.75	15.5 (-5.5 to 10.0)	3.00	14.0 (-6.5 to 7.5)	0.786
AB-MP (degrees)	74.00	42 (49 to 91)	72.00	32 (55 to 87)	0.759
PP-FH (degrees)	2.00	15 (-7 to 8)	3.00	16 (-5 to 11)	0.065
ODI (degrees)	73.50	44 (53 to 97)	74.50	41 (57 to 98)	0.288
FH-NPg (degrees)	87.50	22 (78 to 100)	87.50	17 (70 to 96)	0.761
AB-NPg (degrees)	-7.00	25 (-16 to 9)	-6.50	19 (-14 to 5)	0.557
APDI (degrees)	83.00	45 (65 to 110)	83.00	4162 to 103)	0.965

n = 90. Mann-Whitney U Test. *p* ≤ 0.05.

**Table 3:** Comparison of ODI among overbite groups.

Variables	ODI (degrees)						*P*	Post hoc Dunnet T3		
	Open bite (n = 30)		Normal overbite (n = 30)		Deep bite (n = 30)			Open vs Deep bite (*p*)	Deep vs Normal overbite (*p*)	Open vs Normal overbite (*p*)
	Median	Range	Median	Range	Median	Range				
AB-MP	62.00	31 (49 to 80)	74.50	30 (54 to 84)	81.50	31 (60 to 91)	<0.001**	<0.001**	0.012*	<0.001**
PP-FH	-1.00	14 (-6 to 8)	2.00	16 (-7 to 9)	2.00	18 (-7 to 11)	0.775	0.738	0.963	0.931
ODI	64.50	24 (53 to 77)	74.50	32 (57 to 89)	83.00	42 (56 to 98)	<0.001**	<0.001**	0.022*	<0.001**

n = 90, SD - Standard Deviation. Kruskal-Wallis Test. Post hoc-Dunnet T3. *p ≤ 0.05, **p < 0.01.

Comparison among Angle's classification groups showed significant differences for the facial plane angle (*p* < 0.001), denture base to facial plane angle (p < 0.001) and APDI (*p* < 0.001). However, insignificant difference was found for the palatal plane angle among Classes I, II and III (*p* = 0.214) (Table 4).

**Table 4:** Comparison of APDI among Angle's molar classes groups.

Variables	APDI (degrees)				P		Post hoc Dunnet T3			
	Dental Class I (n = 30)		Dental Class II (n = 30)		Dental Class III (n = 30)			Dental Class I vs II	Dental Class II vs III	Dental Class I vs III
	Median	Range	Median	Range	Median	Range				
FH-NPg	88.00	11 (82 to 93)	85.00	22 (78 to 100)	85.50	18 (79 to 97)	< 0.001**	0.085	0.001*	0.109
PP-FH	3.00	15 (-7 to 8)	-1.00	18 (-7 to 11)	-1.00	15 (-6 to 9)	0.214	0.236	0.999	0.271
AB-NPg	-7.00	13 (-14 to -1)	-9.00	17 (-16 to 1)	1.50	18 (-9 to 9)	<0.001**	0.025*	<0.001**	<0.001**
APDI	83.00	20 (72 to 92)	76.50	31 (62 to 93)	90.50	35 (75 to 110)	<0.001**	0.001*	<0.001**	<0.001**

n = 90, SD - Standard Deviation. Kruskal-Wallis Test. Post hoc-Dunnet T3. *p ≤ 0.05, **p < 0.01.

**Figure 3:**
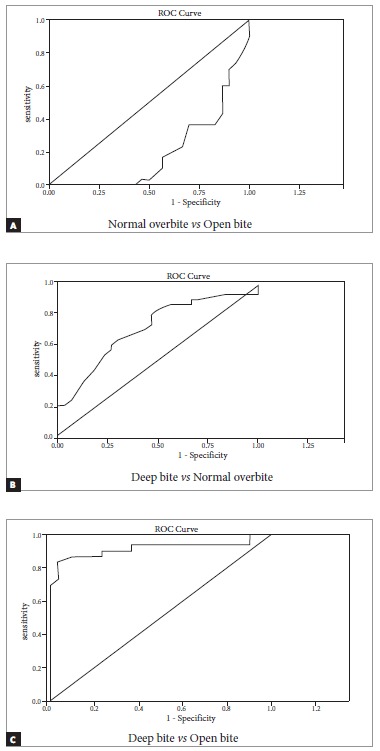
ROC of ODI amongst vertical groups: (A) Normal overbite *vs* Open bite; (B) Deep bite *vs* Normal overbite; (C) Deep bite *vs* Open bite.

ROC plot comparing overbite groups for ODI showed an area under curve with a value equal to 0.196 between normal overbite and open bite groups; 0.70 between deep bite and normal overbite groups; and 0.91 between deep bite and open bite groups. The calculated values of ODI were consistent with incisor overbite in 91% of subjects (Table 5, Fig 3).

**Table 5:** ROC of ODI and APDI among overbite and Angle's classes, respectively.

Study groups	Lower confidence level	Upper confidence level	ROC
ROC of ODI			
*Normal overbite vs Open bite*	0.08	0.30	0.196
*Deep bite vs Normal overbite*	0.57	0.84	0.70
*Deep bite vs Open bite*	0.82	0.99	0.91
ROC of APDI			
*Class I vs II*	0.65	0.90	0.77
*Class I vs III*	0.61	0.86	0.74
*Class II vs III*	0.80	0.97	0.88

ROC = Reciever Operating Characteristic. ROC > 0.6 is significantly reliable.

**Figure 4:**
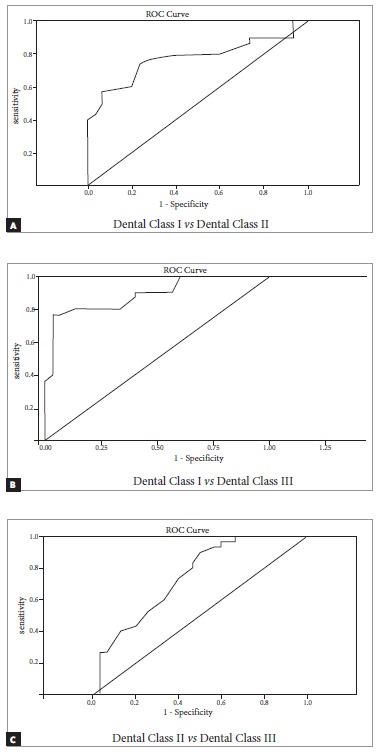
ROC of APDI amongst sagittal groups: (A) Dental Class I *vs* II; (B) Dental Class I *vs* III; (C) Dental Class II *vs* III.

ROC plot comparing Angle's classification groups for APDI showed an area under curve with a value equal to 0.77 between dental Classes I and II; 0.74 between dental Class I and III; and 0.88 between dental Classes II and III. The calculated values of APDI were consistent with Angle's classification in 88% of subjects (Table 5, Fig 4).

## DISCUSSION

Cephalometric analysis is an essential clinical tool in orthodontic diagnosis and treatment planning. To this end, several cephalometric analyses have been introduced by researchers, but none of them provides detailed information regarding dental malocclusion and their corresponding skeletal discrepancy.^6^ Hence, the objective of the current study was to identify whether the skeletal and dental components of malocclusion can be clearly identified by ODI and APDI.

To evaluate ODI, subjects were divided into three equal groups on the basis of overbite. The present study showed significant differences in ODI among open bite, normal overbite and deep bite groups. Our results were in accordance with the study conducted by Kim.^7^ Another study conducted by Freudenthaler et al^12^ found significant differences between deep bite and open bite groups as well as normal overbite and deep bite groups. However, insignificant differences were reported between normal overbite and open bite groups. The reason behind the differences in results may be due to the stratification of subjects on the basis of incisor overbite. 

The ODI is the sum of the AB-MP angle and the palatal plane angle. Considering these components of ODI independently, the AB-MP angle value showed significant intergroup differences. A lower value of AB-MP angle was observed in the horizontal growth pattern, while an increased value was observed in the vertical growth pattern. However, the palatal plane angle did not show significant differences among the three overbite groups. The inclination of palatal plane upward and forward to the Frankfort horizontal plane results in decreased value of ODI. This indicates a tendency towards skeletal open bite. Therefore, it showed that the primary determinant of ODI is AB-MP angle, while the palatal plane angle does not play any significant role in the value of ODI. These results were similar to those reported by other studies.^7^ ^,^ ^12^ ^,^ ^14^

In the assessment of APDI, our study showed significant intergroup differences regarding Angle's classification. Analogous results were found in the previous studies.^11^ ^,^ ^12^ ^,^ ^15^ In evaluating each component individually, the facial plane angle showed significant differences between dental Classes II and III. The lowest values were presented in cases of mandibular retrognathism, while the highest values were found in mandibular excess, indicating skeletal Class III pattern. A higher value of the mean palatal plane angle was observed in dental Class I pattern, but statistical analysis showed insignificant differences among the three Angle's classification groups. In contrast, the third component of APDI, denture base to facial plane angle, showed significant intergroup differences among all three sagittal groups. Clockwise rotation of this angle led to a decrease in the APDI value, which expressed clinically as dental Class II pattern. On the other hand, an increased value of APDI and dental Class III pattern was observed with counter clockwise rotation of this angle.^11^ Hence, facial plane angle and denture base to facial plane angle were the decisive factors for APDI to determine various Angle's classification groups. 

The reliability of diagnostic information provided by the analyses plays a vital role in treatment success. Reliability could be assessed by means of the ROC curve which describes efficacy in terms of sensitivity and specificity.^15^ An ideal test shows a value of 1, while a test result of 0.5 or less indicates no diagnostic value.^16^ ^-^ ^21^ In our study, assessment of ROC demonstrated that ODI yielded the highest diagnostic value for deep bite and open bite groups. These results were 91% correspondent with incisor overbite. In contrast, a study conducted by Freudenthaler et al^12^ reported a value of 81%. Wardlaw et al^20^ showed a high diagnostic value between open bite and positive overbite groups, using a modification of ODI. They used palatal plane to cranial base plane angle (PP-SN) instead of Frankfort horizontal plane to palatal plane angle (PP-FH). Although landmark identification is difficult with the use of the Frankfort horizontal plane, the latter provides more accurate information regarding jaw position. Moreover, a true horizontal plane provides better information in terms of ODI.^7^ ^,^ ^22^

Likewise, applying ROC for APDI demonstrated high diagnostic value among dental Classes II and III malocclusions. These results were in accordance with Angle's classes in 88% of subjects, and a similar value was reported by Freudenthaler et al.^12^ Nevertheless, Kim and Vietas^11^ reported a lower value of 64%. However, there are cases that present different Angle's malocclusions with variable skeletal patterns, i.e., molar Class II could present with skeletal Class III pattern, showing a higher APDI value; or molar Class I with skeletal Class II pattern. Such cases require careful treatment planning and cautious use of biomechanics, since the dental decompensation occurring during orthodontic treatment may result in the expression of underlying skeletal discrepancy. ^23-26^

## CLINICAL IMPLICATION

The results of the present study indicate that ODI and APDI can be reliably used to assess the nature of dental malocclusion. However, there may be cases in which skeletal and dental malocclusions are not in correspondence with each other and for which cautious treatment planning would be required. 

## CONCLUSIONS

The following conclusions could be drawn from this study:

1. No difference was found in ODI and APDI values between males and females, as well as between adolescents and adults.
2. ODI can significantly differentiate between overbite groups and was consistent with patient's overbite in 91% of the cases.
3. APDI can significantly differ between Angle's malocclusions and was consistent with the dental classification in 88% of the cases.
